# Arab ethnicity independently predicts long-term mortality among intensive cardiac care unit admissions

**DOI:** 10.3389/fcvm.2026.1931725

**Published:** 2026-08-05

**Authors:** Itay Torem, Fauzi Shaheen, Louay Taha, Mohammad Karmi, Akiva Brin, Dana Deeb, Pierre Sabouret, Mamas A. Mamas, Asher Schnur, Abed Qadan, Rafel Hitter, Noam Fink, Ari Naimark, Amjad Abu-Salman, Michael Glikson, Elad Asher, For the Jerusalem Platelets Thrombosis Intervention in Cardiology (JUPITER-28) Study Group

**Affiliations:** 1 Jesselson Heart Center, Shaare Zedek Medical Center, the Eisenberg R&D Authority, and Faculty of Medicine, Hebrew University of Jerusalem, Jerusalem, Israel; 2 ACTION Study Group, Institut de Cardiologie, Hôpital Pitié-Salpêtrière, Sorbonne Université, Paris, France; 3 National College of French Cardiologists, Wojo Space, Paris, France; 4 Keele Cardiovascular Research Group, Centre for Prognosis Research, Keele University, Stoke-on-Trent, United Kingdom; 5 Assuta Medical Centers, Tel Aviv, Israel; 6 Department of Cardiothoracic Surgery, Shaare Zedek Medical Center and Faculty of Medicine, Hebrew University of Jerusalem, Jerusalem, Israel

**Keywords:** cardiovascular disease, cardiovascular risk factors, ethnic disparities, health disparities, intensive cardiac care unit (ICCU), long term mortality

## Abstract

**Background:**

Ethnic disparities in cardiovascular health are well recognized worldwide, including in Israel, where Jewish and Arab populations differ in cardiovascular risk profiles and life expectancy. However, data regarding outcomes among critically ill cardiac patients treated in the standardized environment of an intensive cardiac care unit (ICCU) remain limited. This study compared demographic characteristics, comorbidities, clinical presentation, and outcomes between Arab and Jewish patients admitted to a tertiary ICCU.

**Methods:**

We conducted a prospective single-center cohort study including all consecutive patients admitted to the ICCU between July 2019 and December 2025. Patients were stratified by ethnicity (Jewish vs. Arab). The primary endpoint was long-term all-cause mortality. Multivariable Cox regression analysis adjusted for baseline characteristics and index diagnosis.

**Results:**

The cohort included 7,026 patients: 5,615 (79.9%) Jewish and 1,411 (20.1%) Arab. Arab patients were approximately 10 years younger [59.5 (SD 14.9) vs. 69.5 (SD 15.7) years; *p* < 0.001], more often male (77.6% vs. 66.0%; *p* < 0.001), and had a higher prevalence of diabetes mellitus, smoking, dyslipidemia, prior coronary artery disease, and prior coronary artery bypass grafting (all *p* < 0.001). They more frequently presented with ST-elevation myocardial infarction (34.4% vs. 26.7%; *p* < 0.001) and stent thrombosis (1.8% vs. 0.7%; *p* < 0.001). Arab ethnicity was independently associated with higher long-term mortality (adjusted HR 1.31, 95% CI 1.09–1.57; *p* = 0.004).

**Conclusions:**

Despite being younger, Arab patients had a higher burden of cardiovascular risk factors and a 31% increased risk of long-term mortality, highlighting the need for intensified primary and secondary prevention and cardiovascular risk reduction strategies.

## Introduction

Cardiovascular disease (CVD) remains the leading cause of mortality worldwide, accounting for approximately 20 million deaths annually (1–3). Despite continuing advances in prevention and treatment, the global burden of CVD continues to rise, driven primarily by population aging and the persistent prevalence of modifiable cardiovascular risk factors (2, 4).

Cardiovascular outcomes are influenced by both non-modifiable factors including age, sex, genetic predisposition and ethnicity (4, 5), and modifiable risk factors such as hypertension, diabetes mellitus (DM), dyslipidemia, smoking and obesity (4, 5). In recent years, ethnicity has emerged as an independent determinant of cardiovascular risk influencing disease prevalence, age at presentation and clinical outcomes even after adjustment for traditional risk factors (6, 7). These disparities are partly attributable to social and environmental determinants (8).

Israel offers a particularly informative setting for examining ethnic disparities in cardiovascular outcomes. The country has a multiethnic population served by a universal healthcare system (9, 10). Arab citizens, who comprise approximately one-fifth of the population (9), experience higher cardiovascular mortality and shorter life expectancy than Jewish citizens (11, 12). Arab patients also tend to present with acute coronary syndromes (ACS) and heart failure at a younger age and with a heavier burden of modifiable risk factors compared with their Jewish counterparts (13–15).

While ethnic disparities in cardiovascular outcomes have been well documented in population-based and general hospital cohorts (6, 7, 11, 12, 16), far less is known about their persistence among patients admitted to the intensive cardiac care unit (ICCU). The ICCU is a uniquely standardized, protocol-driven environment in which most management decisions follow contemporary guidelines and are delivered by a dedicated multidisciplinary team (17). Examining ethnicity in this setting may therefore help to disentangle baseline patient-level risk from differences in healthcare delivery, and inform strategies aimed at reducing inequalities in cardiovascular care. Accordingly, the present study aimed to describe and compare demographics characteristics, comorbidities, in-hospital course and long-term mortality between Arab and Jewish patients admitted to a tertiary care center ICCU in Jerusalem.

## Materials and methods

### Study overview

This was a single-center, prospective observational cohort study conducted at the ICCU of Shaare Zedek Medical Center, a tertiary referral hospital in Jerusalem admitting more than 1,000 ICCU patients annually. The study was conducted within the framework of the Jerusalem Platelets Thrombosis and Intervention in Cardiology (JUPITER) study group, an institutional ICCU registry and research consortium that has supported multiple investigations of cardiovascular risk factors, treatment patterns, and outcomes in this setting (16, 17).

### Study population

All consecutive patients admitted to the ICCU between July 2019, and December 2025 were eligible for inclusion. Patients were categorized as Jewish or Arab according to ethnicity documented in the electronic medical record. Foreign nationals, tourists, and patients of other or undocumented ethnicities were excluded. For patients with multiple ICCU admissions during the study period, only the first (index) admission was considered; subsequent admissions were not analyzed. The final analytic cohort comprised 7,026 patients (5,615 Jewish and 1,411 Arab).

### Data collection and definitions

Clinical data were prospectively collected during hospitalization by the ICCU coordinator and entered into a structured electronic case report form. Data were checked for accuracy and out-of-range values by the coordinating unit, and any inconsistencies were resolved by review of the source record. Variables captured included demographic characteristics (age, sex, body mass index), cardiovascular comorbidities (hypertension, dyslipidemia, DM, smoking, family history of CAD and other comorbidities), primary diagnosis at ICCU admission, electrocardiographic and echocardiographic data including left ventricular ejection fraction (EF), in-hospital procedures and therapeutic interventions, in-hospital complications and length of stay.

### Ethical approval

The study was approved by the Institutional Review Board of Shaare Zedek Medical Center (IRB protocol number 0052-23-SZMC) and was conducted in accordance with the principles of the Declaration of Helsinki. The requirement for informed consent was waived in light of the observational design and the use of fully de-identified data.

### Outcomes

The primary endpoint was long-term all-cause mortality, defined as death from any cause during the follow-up period. Vital status was ascertained via the Israeli Ministry of Interior population registry. Secondary endpoints were in-hospital mortality, in-hospital complications, and length of ICCU stay. Baseline demographic and clinical characteristics, primary diagnoses, and therapeutic interventions were compared descriptively between groups.

### Statistical analysis

Continuous variables are presented as mean (SD) and were compared between Arab and Jewish patients using Student's t-test, given the large sample size and approximately normal distributions. Categorical variables are presented as counts and percentages and were compared using the chi-square test. Survival was analysed using the Kaplan–Meier method, with differences between ethnic groups assessed by the log-rank test. A multivariable Cox proportional hazards regression model was fitted to identify variables independently associated with long-term mortality. The model included 15 covariates pre-specified on the basis of clinical relevance and the published literature: age, sex, body mass index, ethnicity, hypertension, dyslipidemia, diabetes mellitus, smoking, prior coronary artery disease, chronic renal failure, anemia, ACS presentation, shock at presentation, heart failure/cardiomyopathy presentation, and left ventricular ejection fraction (continuous, %). Analyses were restricted to patients with a complete data set of all model covariates (*n* = 5,447). Missing data primarily involved ejection fraction and BMI measurements. The proportion of missing data was similar between the two ethnic groups, and no imputation was performed. The proportional-hazards assumption was assessed using scaled Schoenfeld residuals, both globally and for each individual covariate. Minor violations of the proportional-hazards assumption were identified for selected covariates; these were minor and were not expected to materially alter the primary findings or their interpretation. Hazard ratios (HR) with 95% confidence intervals (CI) are reported. Given the modest absolute number of in-hospital deaths (*n* = 155, 2.2%) and the absence of a between-group difference in unadjusted analysis, multivariable modelling was not pursued for the in-hospital mortality endpoint. All *p*-values are two-sided, with statistical significance set at *p* < 0.05. Statistical analyses were performed using IBM SPSS Statistics, version 31.0 (IBM Corp., Armonk, NY, USA).

## Results

### Demographic and baseline characteristics

Among the 7,026 patients included in the study, 5,615 (79.9%) were Jewish and 1,411 (20.1%) Arab. Arab patients were approximately ten years younger on average [59.5 (SD 14.9) vs. 69.5 (SD 15.7) years; *p* < 0.001] and were more commonly male (77.6% vs. 66.0%; *p* < 0.001). Body mass index (BMI) was higher in Arab patients [28.7 (SD 5.5) vs. 27.6 (SD 5.2) kg/m²; *p* < 0.001]. Arab patients carried a substantially higher burden of modifiable cardiovascular risk factors including DM (44.7% vs. 34.4%; *p* < 0.001), active or prior smoking (45.1% vs. 21.0%; *p* < 0.001), dyslipidemia (57.6% vs. 51.3%; *p* < 0.001) and family history of coronary artery disease (11.8% vs. 7.5%; *p* < 0.001). Prior coronary artery bypass grafting (CABG) was also more frequent in Arab patients (8.6% vs. 5.7%; *p* < 0.001). Conversely, atrial fibrillation/flutter (12.1% vs. 16.8%), prior transcatheter aortic valve implantation (0.6% vs. 2.2%), cognitive decline (1.8% vs. 4.7%), history of malignancy (5.8% vs. 9.3%) and debilitation (2.1% vs. 4.2%) were more common among Jewish patients (all *p* < 0.001). Moreover, Arab patients had lower prevalence of preserved systolic function (EF ≥50%) compared with Jewish patients (32.9% vs. 46.1%, *p* < 0.001), alongside a higher prevalence of moderately to severely reduced left ventricular systolic function (Table 1).

**Table 1:** Demographic and baseline characteristics of patients.

Variable	Jews (*n* = 5,615)	Arabs (*n* = 1,411)	*p*-value
Age (years)	69.5 (SD 15.7)	59.5 (SD 14.9)	<0.001
Male sex	3,700 (65.9%)	1,094 (77.6%)	<0.001
BMI (kg/m^2^)	27.6 (SD 5.2)	28.7 (SD 5.5)	<0.001
Hypertension	3,353 (59.8%)	818 (58.1%)	0.242
Dyslipidemia	2,880 (51.3%)	813 (57.7%)	<0.001
Diabetes mellitus	1,929 (34.4%)	630 (44.7%)	<0.001
Smoking	1,179 (21.0%)	636 (45.1%)	<0.001
Family history of CAD	420 (7.5%)	166 (11.8%)	<0.001
Prior CAD	1,587 (28.3%)	481 (34.1%)	<0.001
Prior CABG	321 (5.7%)	121 (8.6%)	<0.001
Prior CVA/TIA	436 (7.8%)	88 (6.2%)	0.051
Peripheral arterial disease	252 (4.5%)	57 (4.0%)	0.465
CHF/CMP/MVR	868 (15.5%)	240 (17.0%)	0.151
COPD/asthma	411 (7.3%)	137 (9.7%)	0.003
Pulmonary hypertension	297 (5.3%)	75 (5.3%)	0.966
Prior PE	72 (1.3%)	18 (1.3%)	0.986
Pacemaker/ICD/CRT	349 (6.2%)	93 (6.6%)	0.600
Prior ablation	92 (2.1%)	10 (0.9%)	0.013
Atrial fibrillation/flutter	945 (16.8%)	171 (12.1%)	<0.001
S/p TAVI	126 (2.2%)	9 (0.6%)	<0.001
Cognitive decline	265 (4.7%)	26 (1.8%)	<0.001
Debilitated	238 (4.2%)	30 (2.1%)	<0.001
Malignancy (current/past)	524 (9.3%)	82 (5.8%)	<0.001
Anemia	326 (5.8%)	85 (6.0%)	0.751
CRF	734 (13.1%)	166 (11.8%)	0.191
Dialysis	121 (2.2%)	52 (3.7%)	<0.001
EF			<0.001
Ejection fraction ≤30%	107 (2.0%)	49 (3.7%)	
Ejection fraction 31%–40%	1,190 (22.1%)	377 (28.2%)	
Ejection fraction 41%–49%	1,611 (29.9%)	470 (35.2%)	
Ejection fraction ≥50%	2,486 (46.1%)	440 (32.9%)	

BMI, body mass index; CAD, coronary artery disease; CABG, coronary artery bypass grafting; CVA, cerebrovascular accident; TIA, transient ischemic attack; CHF, congestive heart failure; CMP, cardiomyopathy; MVR, mitral valve replacement; COPD, chronic obstructive pulmonary disease; PE, pulmonary embolism; ICD, implantable cardioverter-defibrillator; CRT, cardiac resynchronization therapy; TAVI, transcatheter aortic valve implantation; CRF, chronic renal failure; EF, ejection fraction.

### Indication for ICCU admission

ACS was the leading reason for ICCU admission in both groups but was significantly more common among Arab patients (59.4% vs. 48.8%; *p* < 0.001), driven primarily by a higher rate of ST-elevation myocardial infarction (STEMI) (34.4% vs. 26.7%; *p* < 0.001) and stent thrombosis (1.8% vs. 0.7%; *p* < 0.001) (Table 2). Peri-procedural myocardial infarction (MI) was also more frequent in Arab patients (2.9% vs. 1.2%; *p* < 0.001). In contrast, Jewish patients more often presented with bradyarrhythmia (9.2% vs. 5.4%; *p* < 0.001), syncope (1.7% vs. 0.8%; *p* = 0.011), pulmonary embolism (PE) (3.9% vs. 2.6%; *p* = 0.013) and post-procedural admissions, particularly post-trans-catheter aortic valve implantation (TAVI) surveillance (11.8% vs. 7.0%; *p* < 0.001). Heart failure or cardiomyopathy as the primary indication was more common in Arab patients (11.7% vs. 9.2%; *p* = 0.011).

**Table 2:** Indication for ICCU admission/primary diagnosis.

Variable	Jews (*n* = 5,615)	Arabs (*n* = 1,411)	*p*-value
ACS (any)	2,737 (48.8%)	837 (59.4%)	<0.001
STEMI (any)	1,500 (26.7%)	484 (34.4%)	<0.001
Acute STEMI	1,239 (22.1%)	391 (27.8%)	<0.001
Stent thrombosis	41 (0.7%)	25 (1.8%)	<0.001
NSTEMI	1,094 (19.5%)	301 (21.4%)	0.117
UAP/chest pain	159 (2.8%)	55 (3.9%)	0.037
Type 2 MI	26 (0.5%)	6 (0.4%)	0.851
Peri-procedural MI	52 (1.2%)	31 (2.9%)	<0.001
Myocarditis	174 (3.1%)	51 (3.6%)	0.324
Takotsubo	72 (1.3%)	18 (1.3%)	0.986
Pulmonary embolism	221 (3.9%)	36 (2.6%)	0.013
SCAD	28 (0.5%)	7 (0.5%)	0.991
Shock (any)	503 (9.5%)	113 (8.6%)	0.325
Cardiogenic shock	365 (6.5%)	81 (5.7%)	0.297
Sepsis	166 (3.0%)	36 (2.6%)	0.417
AF/AFL	91 (2.0%)	10 (0.9%)	0.015
Bradyarrhythmia	518 (9.2%)	76 (5.4%)	<0.001
Syncope	96 (1.7%)	11 (0.8%)	0.011
HF or CMP (any)	418 (9.1%)	130 (11.7%)	0.009
Post-procedural admission (any)	1,052 (21.1%)	189 (15.6%)	<0.001
Post-TAVI	662 (11.8%)	99 (7.0%)	<0.001

ACS, acute coronary syndrome; STEMI, ST-segment elevation myocardial infarction; NSTEMI, non-ST-segment elevation myocardial infarction; UAP, unstable angina pectoris; MI, myocardial infarction; SCAD, spontaneous coronary artery dissection; AF/AFL, atrial fibrillation/atrial flutter; HF, heart failure; CMP, cardiomyopathy; TAVI, transcatheter aortic valve implantation.

### In-hospital management and outcomes

Treatments delivered during the index hospitalization are summarized in Supplementary Table S1. Consistent with the higher prevalence of ACS, Arab patients more frequently underwent diagnostic coronary angiography (14.7% vs. 11.7%; *p* = 0.002) and primary percutaneous coronary intervention (PPCI) (28.2% vs. 24.8%; *p* = 0.010). Pacemaker or implantable cardioverter-defibrillator implantation was more common in Jewish patients (9.3% vs. 6.3%; *p* < 0.001), reflecting the higher prevalence of bradyarrhythmias and structural disease. Rates of mechanical ventilation, vasopressor use, mechanical circulatory support, advanced imaging, and cardiopulmonary resuscitation were comparable between groups.

Overall, in-hospital complication rates (35.3% vs. 33.1%; *p* = 0.17) and length of ICCU stay [2.4 (SD 2.2) vs. 2.4 (SD 2.1) days; *p* = 0.63] were statistically equivalent (Supplementary Table S2).

### Mortality outcomes

In-hospital mortality rates were low and did not differ significantly between Arab and Jewish patients (2.1% vs. 2.2%, respectively; *p* = 0.825).

**Figure 1:**
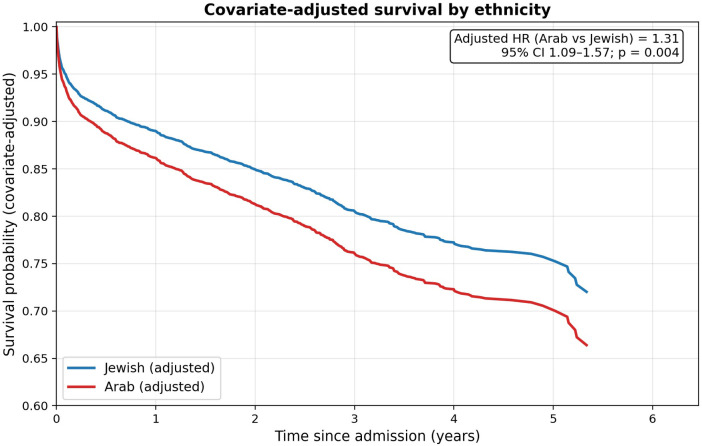
Covariate-adjusted survival by ethnicity.

During a follow-up period of up to 6.5 years, a total of 1,486 deaths occurred (21.2%). After multivariable adjustment from the Cox model, Arab patients exhibited consistently lower adjusted survival than Jewish patients at every time point (adjusted 1-year survival 86.1% vs. 89.0%; adjusted 5-year survival 70.6% vs. 75.7%; Figure 1), corresponding to an adjusted hazard ratio of 1.31 (95% CI 1.09–1.57; *p* = 0.004) and indicating a 31% higher long-term mortality risk independently associated with Arab ethnicity (Supplementary Table S3).

**Figure 2:**
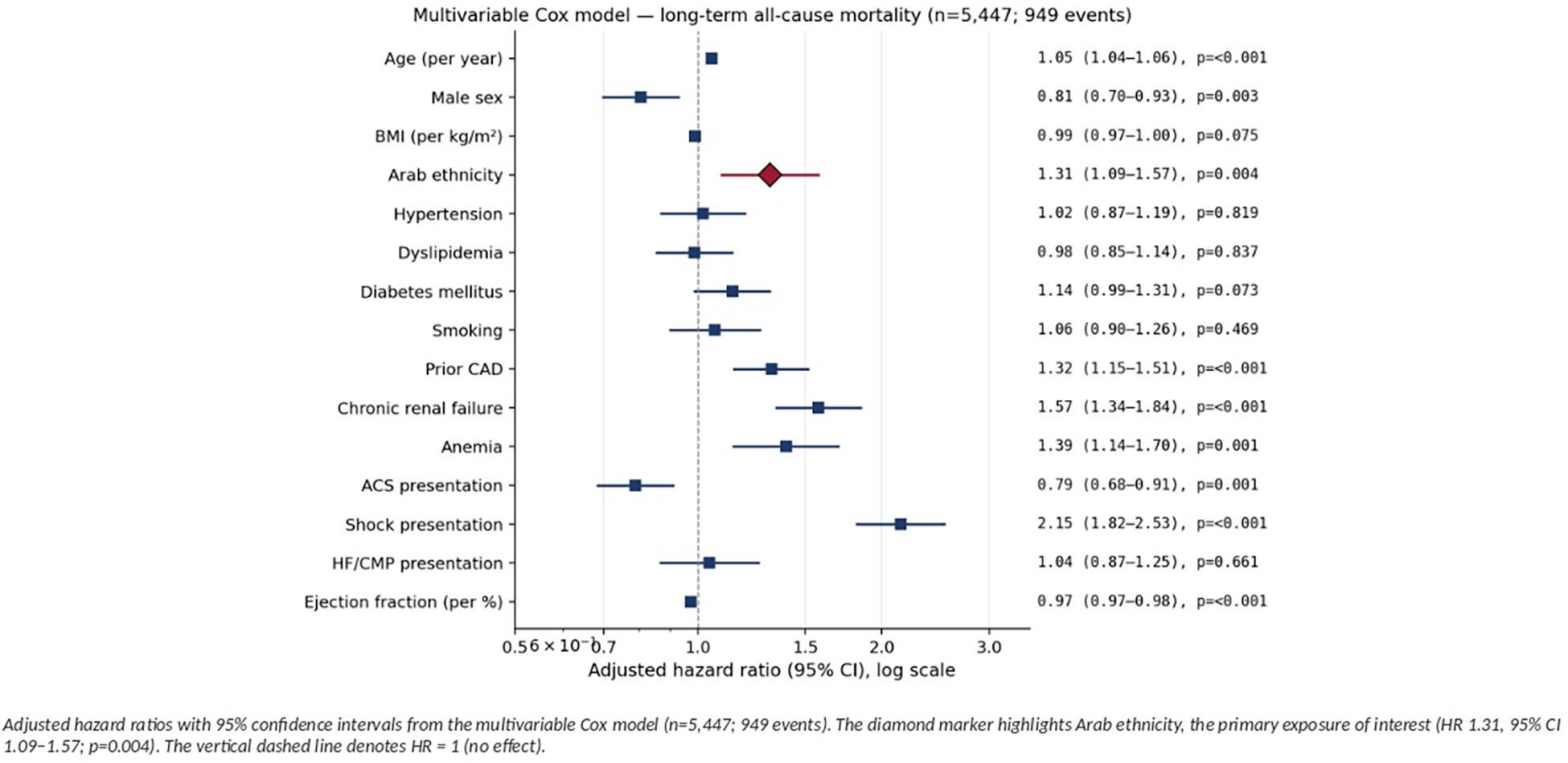
Forest plot of the multivariable Cox proportional hazards model.

Additional independent predictors of higher long-term mortality included older age (HR 1.052 per year, 95% CI 1.045–1.058; *p* < 0.001), cardiogenic shock at presentation (HR 2.15, 95% CI 1.82–2.53; *p* < 0.001), chronic renal failure (CRF) (HR 1.57, 95% CI 1.34–1.85; *p* < 0.001), anemia (HR 1.39, 95% CI 1.14–1.70; *p* = 0.001), and prior CAD (HR 1.32, 95% CI 1.15–1.51; *p* < 0.001). In contrast, each 1% absolute increase in left ventricular ejection fraction was associated with a 2.8% reduction in mortality risk (HR 0.972, 95% CI 0.967–0.977; *p* < 0.001). ACS as the index presentation (HR 0.79, 95% CI 0.68–0.91; *p* = 0.001) and male sex (HR 0.81, 95% CI 0.70–0.93; *p* = 0.003) were independently associated with lower long-term mortality (Figure 2).

### Proportional-hazards diagnostics and sensitivity analysis

The proportional-hazards assumption was satisfied for the primary exposure of interest—ethnicity (Schoenfeld *p* = 0.85), as well as for body mass index, hypertension, dyslipidemia, diabetes mellitus, smoking, prior coronary artery disease, chronic renal failure, anemia, ACS presentation and heart failure/cardiomyopathy presentation (all *p* > 0.05). The assumption was violated for shock at presentation (*p* < 0.001), reflecting its strong early but attenuating late effect on mortality, and was borderline for age (*p* = 0.005), male sex (*p* = 0.031) and ejection fraction (*p* = 0.010). The global Schoenfeld test was significant (*p* < 0.001), driven primarily by shock. In a pre-specified sensitivity analysis restricted to patients without shock at presentation (*n* = 4,944, 751 events), Arab ethnicity remained independently associated with higher long-term mortality (HR 1.23, 95% CI 1.00–1.51; *p* = 0.047), confirming the robustness of the association between Arab ethnicity and increased mortality.

## Discussion

In this large, contemporary, prospective, single-center cohort of 7,026 consecutive ICCU patients, we report three principal findings. First, Arab and Jewish patients presented to the ICCU with markedly different baseline profiles: Arab patients were a decade younger, more often male, and carried a substantially heavier burden of modifiable cardiovascular risk factors, with more frequent STEMI and stent thrombosis. Second, in-hospital management including complications, therapeutic interventions, length of stay, and in-hospital mortality was comparable between groups, consistent with the delivery of equally protocolized contemporary critical cardiac care. Third, and most importantly, although crude long-term mortality appeared numerically lower among Arab patients, this finding was largely attributable to their substantially younger age distribution. After comprehensive multivariable adjustment, Arab ethnicity emerged as an independent predictor of adverse prognosis, conferring a 31% higher risk of long-term all-cause mortality.

The excess long-term mortality among Arab patients despite equivalent in-hospital outcomes most likely reflects a more aggressive cardiovascular risk profile combined with post-discharge disparities in secondary prevention, rather than differences in acute care delivery in the ICCU. Nearly half had DM (44.7%) or were active or prior smokers (45.1%), and over a third had prior CAD (34.1%). These factors, individually and in combination, plausibly accelerate coronary and myocardial disease beyond what cross-sectional adjustment can fully capture. The higher rate of STEMI at admission further suggests greater myocardial injury at the index event, consistent with inadequate risk-factor control in the years preceding presentation (11–13, 15). Additional contributors to the excess long-term mortality may include differences in adherence to secondary prevention treatments, access to outpatient cardiovascular follow-up, and cultural or linguistic barriers to care. Despite comprehensive multivariable adjustment, residual confounding by socioeconomic status, education, medication adherence, and post-discharge healthcare utilization cannot be excluded. Accordingly, the observed association should be interpreted as prognostic rather than causal and should be considered hypothesis-generating, warranting prospective evaluation.

The contrasting Jewish profile was characterized by age-related conditions, including atrial fibrillation/flutter (16.8% vs. 12.1%), malignancy (9.3% vs. 5.8%), and frailty markers such as cognitive decline (4.7% vs. 1.8%) and clinical debilitation (4.2% vs. 2.1%), alongside greater prior exposure to advanced structural procedures, including TAVI (2.2% vs. 0.6%) and catheter ablation (1.6% vs. 0.7%). These findings are consistent with prior studies demonstrating that atrial fibrillation, cancer, frailty, cognitive impairment, and the need for structural cardiac interventions increase markedly with advancing age and are more prevalent in elderly cardiovascular populations. Together, this pattern supports the interpretation that the Jewish ICCU cohort represented an older and more comorbid population profile (18–20). In contrast, Arab patients presented predominantly with STEMI at a younger age, consistent with a heavier modifiable risk-factor burden driving premature myocardial infarction (21). Together, these findings suggest that Arab and Jewish ICCU populations differ not only in risk-factor burden, but also in age-related comorbidities and procedural histories preceding admission.

### Limitations

Several limitations should be acknowledged. First, this was a single-center study conducted at a tertiary referral center in Jerusalem, which may limit the generalizability of the findings to other geographic regions in Israel or non-tertiary healthcare settings. Second, as with all observational studies, residual and unmeasured confounding cannot be excluded, particularly regarding socioeconomic factors, adherence to guideline-directed medical therapy after discharge, and access to outpatient cardiovascular follow-up. Third, the primary endpoint was all-cause mortality rather than cardiovascular mortality, precluding differentiation between cardiovascular and non-cardiovascular causes of death contributing to the excess risk observed among Arab patients. Finally, Jewish patients were analyzed as a single ethnic group without subclassification by ancestral origin. The Israeli Jewish population is ethnically heterogeneous, comprising Ashkenazi, Mizrahi, Ethiopian, and former Soviet Union immigrant subgroups, which may differ in cardiovascular risk profiles and long-term outcomes as shown in past studies (22). Future studies should stratify Jewish patients by ancestral subgroup to more precisely characterize the independent role of ethnicity in cardiovascular prognosis.

## Conclusions

Among patients admitted to a contemporary tertiary ICCU in Jerusalem, Arab patients presented at a significantly younger age but with a substantially higher burden of modifiable cardiovascular risk factors and more frequent STEMI presentation. Despite similar in-hospital management and short-term outcomes, Arab ethnicity was independently associated with a 31% increase in long-term mortality after multivariable adjustment. Multicenter studies and access to socioeconomic, adherence and outpatient-follow-up data are needed to confirm these findings and to identify the modifiable determinants that registry-based analyses alone cannot capture.

## Data Availability

The data analyzed in this study is subject to the following licenses/restrictions: Information can be accessed upon contacting the lead author and obtaining approval from the Shaare Zedek institution. Requests to access these datasets should be directed to Itay Torem, itay.torem@mail.huji.ac.il.
