# Supplementary material for: Arab ethnicity independently predicts long-term mortality among intensive cardiac care unit admissions
Source: Front Cardiovasc Med. 2026 Aug 5;13:1931725. doi: 10.3389/fcvm.2026.1931725 (PMC13485772; doi:10.3389/fcvm.2026.1931725)
Supplement: Supplementary file 1 [file Table1.pdf]

Supplementary Table S1. Treatments delivered during the index ICCU hospitalization

Variable	Jews (n=5,615)	Arabs (n=1,411)	p-value
Coronary angiography (Cath.)	656 (11.7%)	207 (14.7%)	0.002
PCI	900 (16.0%)	248 (17.6%)	0.157
Primary PCI (PPCI)	1392 (24.8%)	397 (28.2%)	0.010
CABG	117 (2.1%)	39 (2.8%)	0.121
VT ablation	38 (0.7%)	11 (0.8%)	0.675
EPS / ablation	55 (1.0%)	19 (1.4%)	0.225
Pacemaker/AICD	520 (9.5%)	86 (6.3%)	<0.001
DC cardioversion	140 (2.6%)	35 (2.6%)	0.984
Structural intervention (any)	125 (2.9%)	27 (2.6%)	0.620
TAVI	35 (0.8%)	4 (0.4%)	0.147
Thrombolysis / Inari	118 (2.1%)	32 (2.3%)	0.698
Pericardiocentesis	121 (2.2%)	17 (1.2%)	0.022
Peripheral intervention & stent	27 (0.6%)	9 (0.8%)	0.395
Desensitization	148 (3.3%)	45 (4.2%)	0.161
CPR	293 (5.2%)	81 (5.7%)	0.433
Mechanical ventilation	420 (7.5%)	101 (7.2%)	0.682
Tracheostomy	18 (0.3%)	10 (0.7%)	0.038
Dialysis	93 (1.7%)	25 (1.8%)	0.762
A-line	885 (15.8%)	226 (16.0%)	0.810
Vasopressors	687 (12.2%)	178 (12.6%)	0.694
MCS (any)	233 (5.2%)	62 (5.7%)	0.469
TTM (hypothermia)	89 (1.6%)	32 (2.3%)	0.078
Advanced imaging (any)	619 (11.0%)	150 (10.6%)	0.675

Abbreviations: Cath., coronary angiography (catheterization); PCI, percutaneous coronary intervention; PPCI, primary percutaneous coronary intervention; CABG, coronary artery bypass grafting; VT, ventricular tachycardia; EPS, electrophysiologic study; AICD, automatic implantable cardioverter-defibrillator; DC, direct current; CPR, cardiopulmonary resuscitation; A-line, arterial line; MCS, mechanical circulatory support; TTM, targeted temperature management.
