# Supplementary material for: Arab ethnicity independently predicts long-term mortality among intensive cardiac care unit admissions
Source: Front Cardiovasc Med. 2026 Aug 5;13:1931725. doi: 10.3389/fcvm.2026.1931725 (PMC13485772; doi:10.3389/fcvm.2026.1931725)
Supplement: Supplementary file 2 [file Table2.pdf]

Supplementary Table S2. In-hospital complications, mortality, and length of stay

Variable	Jews (n=5,615)	Arabs (n=1,411)	p-value
In-hospital complication (any)	1491 (33.1%)	381 (35.3%)	0.180
Malignant arrhythmia	99 (1.8%)	35 (2.5%)	0.078
Shock	190 (3.4%)	42 (3.0%)	0.445
CHF exacerbation	131 (2.3%)	44 (3.1%)	0.090
Mechanical complications (VSR/rupture)	44 (0.8%)	12 (0.9%)	0.800
LV thrombus	74 (1.4%)	17 (1.2%)	0.740
Sepsis	110 (2.0%)	35 (2.5%)	0.217
Stroke/TIA	66 (1.2%)	10 (0.7%)	0.130
Re-infarction	33 (0.6%)	8 (0.6%)	0.928
Stent thrombosis	727 (17.0%)	201 (19.6%)	0.049
ARF	193 (3.4%)	53 (3.8%)	0.559
Significant bleeding	195 (3.5%)	41 (2.9%)	0.291
Blood transfusion	231 (5.2%)	41 (3.8%)	0.064
Vascular complications	104 (2.3%)	20 (1.9%)	0.350
Anoxic brain damage	24 (0.5%)	10 (0.9%)	0.139
In-hospital death	125 (2.2%)	30 (2.1%)	0.825
Overall death during follow-up	1211 (21.6%)	275 (19.6%)	0.092
Length of stay (days)	2.4 ± 2.1	2.4 ± 2.2	0.619

Abbreviations: VSR, ventricular septal rupture; LV, left ventricular; ARF, acute renal failure; TIA, transient ischemic attack; SD, standard deviation.
