# Supplementary material for: Arab ethnicity independently predicts long-term mortality among intensive cardiac care unit admissions
Source: Front Cardiovasc Med. 2026 Aug 5;13:1931725. doi: 10.3389/fcvm.2026.1931725 (PMC13485772; doi:10.3389/fcvm.2026.1931725)
Supplement: Supplementary file 3 [file Table3.pdf]

Supplementary Table S3. Multivariable Cox proportional hazards model predictors of long-term mortality

Variable	Adjusted HR	95% CI	p-value
Age (per year)	1.052	1.045 – 1.058	<0.001
Male sex	0.805	0.699 – 0.928	0.003
BMI (per kg/m²)	0.988	0.975 – 1.001	0.075
**Arab ethnicity**	1.310	1.092 – 1.572	0.004
Hypertension	1.019	0.870 – 1.192	0.819
Dyslipidemia	0.985	0.855 – 1.135	0.837
Diabetes mellitus	1.137	0.988 – 1.308	0.073
Smoking	1.065	0.899 – 1.261	0.469
Prior CAD	1.318	1.146 – 1.514	<0.001
Chronic renal failure	1.574	1.344 – 1.845	<0.001
Anemia	1.392	1.142 – 1.696	0.001
ACS presentation	0.788	0.684 – 0.908	0.001
Shock presentation	2.148	1.822 – 2.532	<0.001
HF/CMP presentation	1.042	0.867 – 1.253	0.661
Ejection fraction (per %)	0.972	0.967 – 0.977	<0.001

Abbreviations: HR, hazard ratio; CI, confidence interval; BMI, body mass index; CAD, coronary artery disease; ACS, acute coronary syndrome; HF, heart failure; CMP, cardiomyopathy. Reference categories: Jewish ethnicity; female sex; non-ACS presentation; non-shock presentation; non-HF/CMP presentation. Arab ethnicity row highlighted for emphasis.
